# Protective Effect of CXCR3^+^CD4^+^CD25^+^Foxp3^+^ Regulatory T Cells in Renal Ischemia-Reperfusion Injury

**DOI:** 10.1155/2015/360973

**Published:** 2015-07-27

**Authors:** Cao Jun, Li Qingshu, Wei Ke, Li Ping, Dong Jun, Luo Jie, Min Su

**Affiliations:** ^1^Department of Anesthesiology, The First Affiliated Hospital of Chongqing Medical University, No. 1 Youyi Road, Yuzhong District, Chongqing 400016, China; ^2^Department of Pathology, Chongqing Medical University, Chongqing 400016, China

## Abstract

Regulatory T cells (Tregs) suppress excessive immune responses and are potential therapeutic targets in autoimmune disease and organ transplantation rejection. However, their role in renal ischemia-reperfusion injury (IRI) is unclear. Levels of Tregs and expression of CXCR3 in Tregs were analyzed to investigate their function in the early phase of renal IRI. Mice were randomly divided into Sham, IRI, and anti-CD25 (PC61) + IRI groups. The PC61 + IRI group was established by i.p. injection of PC61 monoclonal antibody (mAb) to deplete Tregs before renal ischemia. CD4^+^CD25^+^Foxp3^+^ Tregs and CXCR3 on Tregs were analyzed by flow cytometry. Blood urea nitrogen (BUN), serum creatinine (Scr) levels, and tubular necrosis scores, all measures of kidney injury, were greater in the IRI group than in the Sham group. Numbers of Tregs were increased at 72 h after reperfusion in kidney. PC61 mAb preconditioning decreased the numbers of Tregs and aggravated kidney injury. There was no expression of CXCR3 on Tregs in normal kidney, while it expanded at 72 h after reperfusion and inversely correlated with BUN, Scr, and kidney histology score. This indicated that recruitment of Tregs into the kidney was related to the recovery of renal function after IRI and CXCR3 might be involved in the migration of Tregs.

## 1. Introduction

Ischemia-reperfusion injury (IRI) is a common and important clinical problem in many different organs. It has a critical role in the pathogenesis of acute renal failure and graft rejection, is associated with increased morbidity and mortality, and is closely related to the development of chronic kidney disease [1, 2].

Increasing numbers of studies implicate important roles for immune and inflammatory pathways in IRI [3, 4]. The activation and accumulation of neutrophils and macrophages in the innate immune phase had been thought to be the prime cellular mediator of microvascular plugging and local tissue damage in the IRI model [5]. There was a viewpoint that both T and B cells constituted the primary mediators of the adaptive immune response and did not play a role in the acute phase of IRI. However, recent data have challenged this assumption and demonstrate an important modulatory role of T cells in IRI [6–8].

Regulatory T cells (Tregs), a subset of CD4^+^T cells, suppress excessive immune responses. This population of cells is commonly identified by their expression of CD4 and CD25 on the cell surface and their upregulation of the transcription factor forkhead box P3 (FoxP3) [9]. Multiple mechanisms of action for Tregs have been reported [10], such as direct cell-cell contact, depletion of interleukin- (IL-) 2, release of soluble inhibitory factors like IL-10 or transforming growth factor-*β*, and hydrolysis of ATP to adenosine, which exhibit direct immunosuppressive effects. Furthermore, Tregs have the ability to traffic to areas of inflammation to regulate immune reactions [11]. Previous studies have demonstrated that the chemokine receptor CXCR3 plays an important role in Treg cell recruitment in transplantation rejection [12] and inflammatory reactions [13]. In the present study, the levels of Tregs and the expression of CXCR3 on Tregs were measured to investigate the role of CXCR3^+^ Tregs in renal IRI.

## 2. Materials and Methods

### 2.1. Animals

Male C57BL/6J mice, aged 8 to 12 weeks and weighing 20 to 25 g, were obtained from the Animal Center of Chongqing Medical University. One week before the experiments, animals were housed in a specific pathogen-free laboratory in an acclimatized room at standard room conditions (25°C, 55% humidity) with regular 12 h/12 h light/dark cycles. They were fed with a standard diet and had free access to tap water. All experiments were conducted in accordance with the Declaration of Helsinki (1964) and the “Principles of Laboratory Animal Care” NIH Publication vol. 25, no. 28, revised 1996. The study was performed under the Reduction, Replacement, and Refinement principle. All experimental procedures involving animals were approved by the Animal Ethics and Use Committee of Chongqing Medical University.

### 2.2. Experimental Groups

Mice were randomly divided into three groups: Sham (*n* = 16), IRI (*n* = 16), and PC61 + IRI (*n* = 16), where PC61 is a monoclonal antibody (mAb) to CD25. It has been reported that PC61 has no effect on renal function in normal or Sham mice [14], which we confirmed in a preliminary study. Thus, we did not have an additional experimental group in which Sham mice were administered the mAb.

#### 2.2.1. Sham Group

Sham animals underwent the same surgical procedure without clamping of the renal pedicles. Eight mice were sacrificed at 24 h and 72 h after the operation.

#### 2.2.2. IRI Group

Microvascular clamps were placed on both renal pedicles for 45 min. The clamps were removed, and the wounds were sutured. Eight mice were sacrificed at 24 h and 72 h after IRI.

#### 2.2.3. PC61 + IRI Group

Depletion of Tregs was performed by i.p. injection with 250 *μ*g of anti-CD25 (PC61) mAb (eBioscience; San Diego, CA, USA) at 24 h before renal ischemia [15]. Eight mice were sacrificed at 24 h and 72 h after IRI.

### 2.3. Experimental Protocol

The animals were anaesthetized with i.p. administration of 10% ketamine mixed with 2% xylazine. The renal pedicles were bluntly dissected. Microvascular clamps were placed on both renal pedicles for 45 min. The clamps were removed, and the wounds were sutured. The animals were placed on a heating pad and monitored visually until they were completely awake. After the operation, the animals were left to recover at room temperature, with immediate and unrestricted access to food and water. In the first 24 h after the surgery, the animals were treated with 2 *μ*g of tramadol hydrochloride per g of body weight every 8 h for postoperative analgesia. Mice were sacrificed at 24 h and 72 h after IRI, and blood samples were collected from the inferior vena cava. Blood urea nitrogen (BUN) and serum creatinine (Scr) levels were measured to evaluate renal function. Half of the left kidney was dissected at each time point for histology assessment. The other half of the left kidney and the right kidney were homogenized, and the leukocytes within the tissue were isolated. The numbers of CD4^+^CD25^+^Foxp3^+^ Tregs and CXCR3^+^CD4^+^CD25^+^Foxp3^+^ Tregs in kidney tissue were analyzed by flow cytometry. The correlation of these populations with BUN, Scr, and tubular necrosis score was calculated.

### 2.4. BUN and Scr Measurements

Whole blood (0.6 mL) was collected and the serum was isolated for BUN and Scr measurements with a Hitachi 747 automatic analyzer (Hitachi; Tokyo, Japan).

### 2.5. Flow Cytometry

Kidney tissue was disrupted mechanically in 10 mL of RPMI 1640 medium supplemented with 5% of newborn calf serum using a homogenizer. To remove debris, samples were passed through a 100 *μ*m cell strainer with 50 mL of RPMI 1640 medium at 4°C. Leukocytes were isolated by centrifugation-suspension in Percoll at 4°C. Multiple-color immunofluorescence staining was analyzed using a FACS flow cytometer (FACSCalibur; BD Biosciences; Franklin Lakes, NJ, USA). The fluorochrome-conjugated monoclonal antibodies anti-CD4 Percp (RM 4-5), anti-CXCR3 PE (1C6/CXR3), anti-CD25 FITC (PC61.5), and anti-Foxp3 Alexa 647 (259D/C7) were purchased from eBioscience. Surface staining was used for detection of CD4, CD25, and CXCR3 whereas intracellular staining was used for detection of Foxp3 at 4°C. Data were analyzed by using FlowJo for Windows (Version 7.2.5; Ashland, OR, USA).

### 2.6. Kidney Histology

Paraformaldehyde-fixed and paraffin-embedded 4 *μ*m sections were stained with hematoxylin-eosin (H&E). Damaged tubules were identified by the presence in the cortex of diffuse tubular dilatation, intraluminal casts, and/or tubular cell blebbing, vacuolization, and detachment, as assessed in a blinded fashion by a renal pathologist in ten high-power fields (400x magnification) per section. The percentages of histological changes in the kidney tissue were scored using a semiquantitative scale designed to evaluate the degree of tubular necrosis as follows [16, 17]: 0 = normal kidney; 1 = minimal necrosis (5% involvement); 2 = mild necrosis (5–25% involvement); 3 = moderate necrosis (25–50% involvement); 4 = severe necrosis (50–75% involvement); and 5 = most severe necrosis (>75% involvement).

### 2.7. Statistical Analysis

Comparisons between multiple groups were performed by a one-way analysis of variance test followed by the Kruskal-Wallis test where appropriate. Significance for differences between independent groups was determined using the Mann-Whitney *U* test. Spearman's rank correlation was applied for detecting correlation between different study parameters. Two-tailed *P* values < 0.05 were considered to be significant. For statistical analyses, GraphPad Prism version 5.0 was used (GraphPad Software; San Diego, CA, USA).

## 3. Results

### 3.1. Renal Function Assessment after IRI

**Figure 1:**
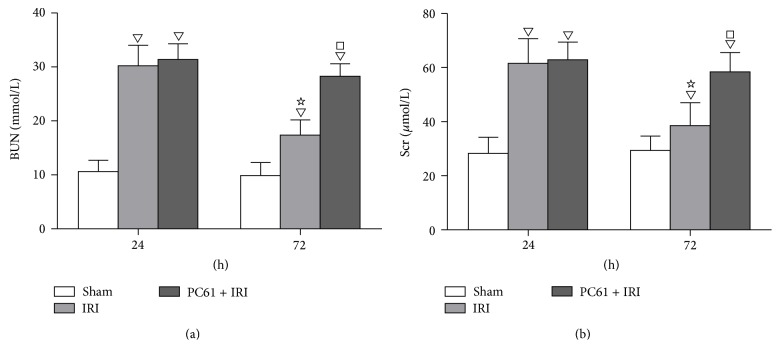
BUN and Scr levels at 24 h and 72 h after reperfusion. (a) Concentrations of BUN. (b) Concentrations of Scr. Values of the bar graphs represent the mean ± SD (*n* = 8 per group). Compared with the Sham group, ^▽^ *P* < 0.05; compared with the IRI group at 24 h after reperfusion, ^☆^ *P* < 0.05; compared with the IRI group at 72 h after reperfusion, ^□^ *P* < 0.05.

BUN and Scr levels were both greater in the IRI group than in the Sham group at 24 h (30.2 ± 3.8 mmol/L* versus *10.6 ± 2.1 mmol/L and 61.6 ± 9.1 *μ*mol/L* versus *28.3 ± 5.9 *μ*mol/L, resp., *P* < 0.05) and at 72 h (17.4 ± 2.8 mmol/L* versus *9.9 ± 2.4 mmol/L and 38.6 ± 8.4 *μ*mol/L* versus *29.4 ± 5.3 *μ*mol/L, resp., *P* < 0.05) after reperfusion. However, the BUN and Scr concentrations were decreased at 72 h compared with those at 24 h in the IRI group (*P* < 0.05), although both concentrations were still greater than those in the Sham group (*P* < 0.05). With PC61 mAb administration before renal ischemia, BUN and Scr levels were greater at 72 h (28.3 ± 2.3 mmol/L* versus *17.4 ± 2.8 mmol/L and 58.4 ± 7.2 *μ*mol/L* versus *38.6 ± 8.4 *μ*mol/L, resp., *P* < 0.05) but not at 24 h after reperfusion as compared with the IRI group (Figure 1).

### 3.2. Kidney Histology

**Figure 2:**
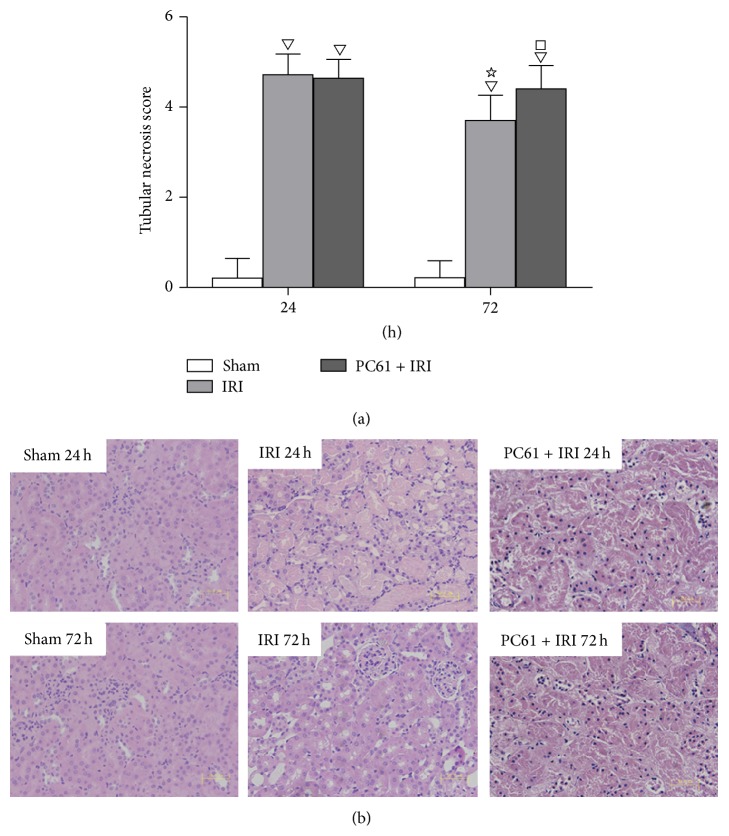
Renal histology at 24 h and 72 h after reperfusion. (a) Tubular necrosis scores in each group. Values of the bar graphs represent the mean ± SD (*n* = 8 per group). Compared with the Sham group, ^▽^ *P* < 0.05; compared with the IRI group at 24 h after reperfusion, ^☆^ *P* < 0.05; compared with the IRI group at 72 h after reperfusion, ^□^ *P* < 0.05. (b) Representative histology images of H&E stained renal sections at 24 h and 72 h after reperfusion (original magnification: ×400, scale bar: 50 *μ*m). After IRI, a large number of tubular epithelial cells swelled with vacuolar degeneration, parts of the cells shrunk and had dark nuclei, parts of the cells lost intracellular structure due to cellular necrosis and abscission, and cellular debris and casts were seen in the enlarged lumen.

Kidney tissue structure was normal in the Sham group, with only a few swollen renal tubular epithelial cells. After IRI or PC61 treatment, a large number of tubular epithelial cells swelled, parts of the cells shrunk and had dark nuclei, the basal membrane was fractured due to cell necrosis and abscission, and cellular debris and casts were seen in the enlarged lumen. The tubular necrosis score in the IRI group was greater than that in the Sham group at 24 h and 72 h after reperfusion (*P* < 0.05). With PC61 mAb administration before renal ischemia, the score was greater at 72 h (4.25 ± 0.46* versus *3.70 ± 0.56, *P* < 0.05) but not at 24 h as compared with the IRI group (Figure 2).

### 3.3. Changes of CD4^+^CD25^+^Foxp3^+^ Tregs and CXCR3^+^CD4^+^CD25^+^Foxp3^+^ Tregs in the Kidney after Reperfusion

**Figure 3:**
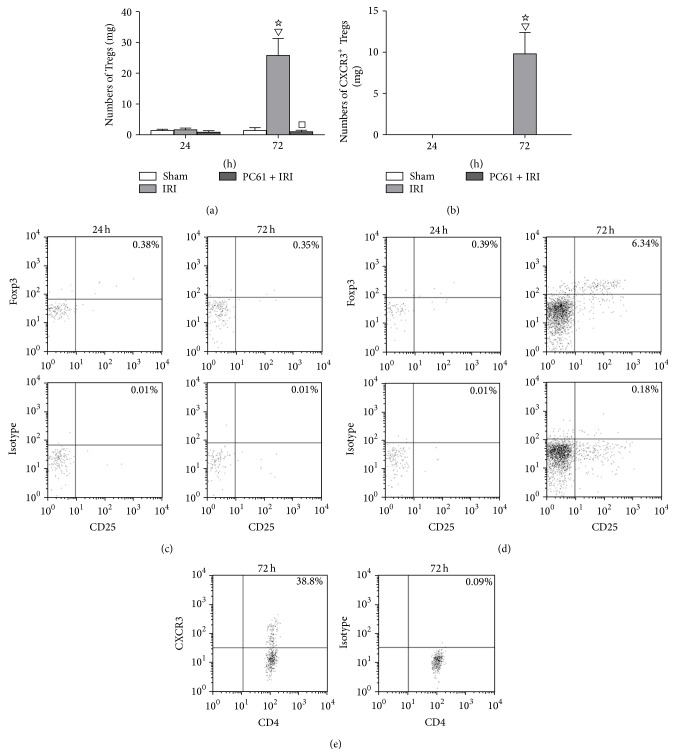
Numbers of Tregs and CXCR3^+^ Tregs in kidney at 24 h and 72 h after reperfusion measured by flow cytometry analysis. (a) Numbers of CD4^+^CD25^+^Foxp3^+^ Tregs in kidney at 24 h and 72 h after reperfusion. (b) Numbers of CXCR3^+^CD4^+^CD25^+^Foxp3^+^ Tregs in kidney at 24 h and 72 h after reperfusion. Values of the bar graphs represent the mean ± SD (*n* = 8 per group). Compared with the Sham group, ^▽^ *P* < 0.05; compared with the IRI group at 24 h after reperfusion, ^☆^ *P* < 0.05; compared with the IRI group at 24 h or 72 h after reperfusion, ^□^ *P* < 0.05. (c) Representative dot plots of Tregs in kidney from Sham group at 24 h and 72 h after reperfusion. (d) Representative dot plots of Tregs in kidney from IRI group at 24 h and 72 h after reperfusion. The CD4^+^ T cells were initially gated from the lymphocyte area by forward scatter (FSC)* versus* side scatter (SSC), and then the expression of CD25 and Foxp3 was analyzed in this population. The percentage of CD4^+^CD25^+^Foxp3^+^ Tregs in CD4^+^ cells was obtained in the upper right quadrant. (e) Representative dot plots of CXCR3^+^ Tregs from the IRI group. Because there were no CXCR3^+^ Tregs detected in the kidney from the Sham or IRI group at 24 h after IRI, only dot plots from the IRI group at 72 h after reperfusion were shown. The percentage of CXCR3 in the CD4^+^CD25^+^Foxp3^+^ population was shown in the upper right quadrant.

There was almost no detectable expression of CD4^+^CD25^+^Foxp3^+^ Tregs in kidney from the Sham group or IRI group at 24 h after reperfusion. The numbers of CD4^+^CD25^+^Foxp3^+^ Tregs were increased more than 20-fold at 72 h in the IRI group as compared with the Sham group or that at 24 h in the IRI group (*P* < 0.05). The administration of PC61 mAb depleted Tregs in the kidneys (Figure 3(a)).

There was no detectable expression of CXCR3^+^CD4^+^CD25^+^Foxp3^+^ Tregs in the kidney from the Sham group or the IRI group at 24 h after reperfusion. The numbers of CXCR3^+^CD4^+^CD25^+^Foxp3^+^ Tregs were increased more than 10-fold at 72 h in the IRI group as compared with the Sham group or 24 h in the IRI group (*P* < 0.05). Administration of PC61 mAb effectively depleted the above population of cells in the kidneys (Figure 3(b)).

Representative dot plots of CD4^+^CD25^+^Foxp3^+^ Tregs in the kidney from the Sham group and the IRI group were shown in Figures 3(c) and 3(d). There was almost no detectable expression of CD4^+^CD25^+^Foxp3^+^ Tregs in the Sham group or at 24 h in the IRI group. In contrast, the percentage of this population in CD4^+^ T cells in the IRI group expanded significantly at 72 h.

Because there were no CXCR3^+^CD4^+^CD25^+^ Tregs detected in the kidney from the Sham group or IRI group at 24 h, only the dot plots from the IRI group at 72 h after reperfusion were shown in Figure 3(e). The proportion of CXCR3^+^CD4^+^CD25^+^ Tregs in CD4^+^CD25^+^Foxp3^+^ Tregs markedly increased at 72 h after reperfusion.

### 3.4. Correlation of CD4^+^CD25^+^Foxp3^+^ Tregs and CXCR3^+^CD4^+^CD25^+^Foxp3^+^ Tregs with Kidney Injury in the IRI Group

**Figure 4:**
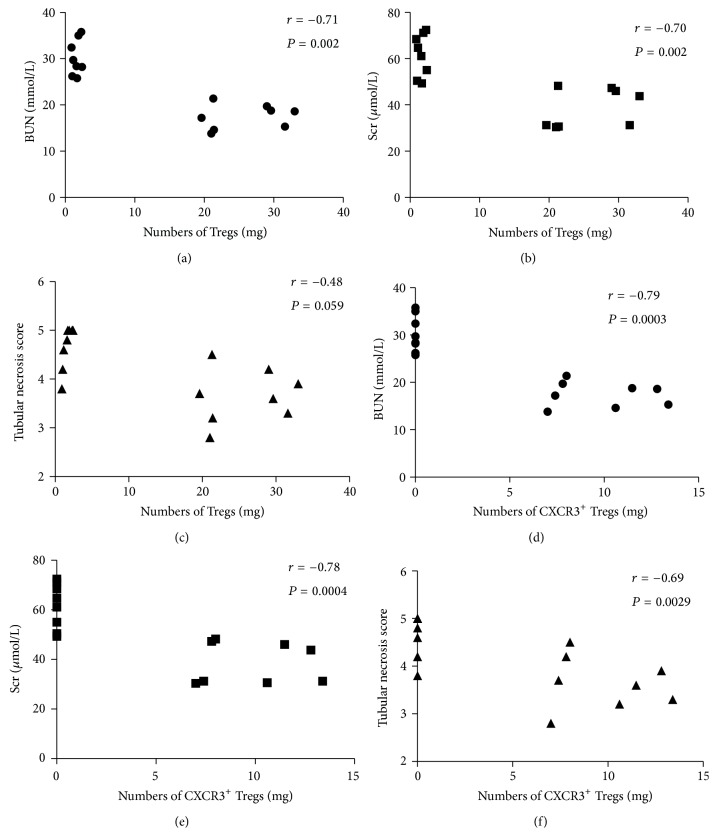
Correlation of CD4^+^CD25^+^Foxp3^+^ Tregs and CXCR3^+^CD4^+^CD25^+^Foxp3^+^ Tregs with BUN, Scr, and tubular necrosis score in the IRI group. The numbers of CD4^+^CD25^+^Foxp3^+^ Tregs in the kidney were negatively correlated with BUN (a) and Scr (b) but not tubular necrosis score (c). The numbers of CXCR3^+^CD4^+^CD25^+^Foxp3^+^ Tregs in the kidney were negatively correlated with BUN (d), Scr (e), and tubular necrosis score (f). Each symbol represents a single individual (*n* = 16).

Numbers of CD4^**+**^CD25^**+**^Foxp3^**+**^ Tregs in the kidney from the IRI group were negatively correlated with BUN (*r* = −0.71, *P* < 0.05) and Scr (*r* = −0.70, *P* < 0.05), while the relationship with tubular necrosis score was not clear (*r* = −0.48, *P* > 0.05) (Figures 4(a), 4(b), and 4(c)).

Numbers of CXCR3^**+**^CD4^**+**^CD25^**+**^Foxp3^**+**^ Tregs in the kidney from the IRI group were negatively correlated with BUN (*r* = −0.79, *P* < 0.05), Scr (*r* = −0.78, *P* < 0.05), and tubular necrosis score (*r* = −0.69, *P* < 0.05) (Figures 4(d), 4(e), and 4(f)).

## 4. Discussion

In the present study, we observed the protective effect and migratory phenomenon of Tregs in the kidney after IRI. BUN, Scr levels, and tubular necrosis scores, all potential measures of kidney injury, were greater in the IRI group than in the Sham group. Numbers of Tregs were increased at 72 h after reperfusion in the kidney. PC61 mAb preconditioning decreased the numbers of Tregs and aggravated kidney injury. There were no CXCR3^**+**^ Tregs in normal kidneys, while this population expanded at 72 h after reperfusion and was inversely correlated with BUN, Scr, and kidney histology score.

Tregs are a subset of helper T cells and have potent immunosuppressive effects. CD25 expression on T lymphocytes is upregulated by antigenic or mitogenic stimulation [18–20]. Soluble CD25/IL-2R*α* is produced as a consequence of lymphocyte stimulation and is found in biological fluids following inflammatory responses. As nuclear transcription factor Foxp3 has been shown not only to represent a specific marker for CD4^+^CD25^+^Tregs but also to regulate their development and function [21, 22], it is currently the most accepted marker for identification of Tregs. Thus, CD4^**+**^CD25^**+**^Foxp3^**+**^ T cells were defined as Tregs in our study.

Few Tregs were observed in kidneys from normal mice or mice in the IRI group at 24 h after reperfusion, while their numbers significantly increased at 72 h after reperfusion. The BUN and Scr levels as well as the tubular necrosis score reflected kidney damage after IRI. These results showed that significant renal injury occurred at 24 h after reperfusion and that there was gradual recovery at 72 h. It is reasonable to conclude that the increasing numbers of Tregs had an effect on improvement of renal function after reperfusion. To validate this hypothesis, we used PC61 to investigate this protective effect.

A rat IgG1 anti-mouse-CD25 (IL-2R*α*) mAb called PC61 has been shown to selectively deplete CD4^+^CD25^+^ T cells* in vivo*, and this depletion was reversible within a few days [23, 24]. In a preliminary study, we tested the effect of the PC61 mAb and found that it depleted almost all Tregs in both Sham and IRI mice by intraperitoneal injection. Hence, the PC61 mAb was used to abolish the effect of Tregs and investigate its function in IRI. We found that depletion of Tregs with an anti-CD25 mAb potentiated kidney damage at 72 h after reperfusion. These results proved Tregs had protective effects on the kidneys after IRI. Using a murine model of ischemic acute kidney injury, Gandolfo et al. [25] found that there was significant trafficking of Tregs into the kidneys from 3 to 10 days after reperfusion. The infusion of Tregs after IRI had minimal effects on neutrophil and macrophage infiltration but had a late beneficial effect on kidney repair, likely through the modulation of proinflammatory cytokine production of other T-cell subsets. This result showed the important role of Tregs in the repair of ischemic acute kidney injury. Key issues to address include understanding why there are almost no Tregs in normal kidneys and when and how did these cells appear in the inflammatory tissue.

CXCR3 is an important chemokine receptor activated by three interferon-inducible ligands CXCL9, CXCL10, and CXCL11. Early studies demonstrated a role for CXCR3 in the trafficking of Th1 and CD8 T cells to peripheral sites of Th1-type inflammation and the establishment on Th1 amplification loop mediated by IFN*γ* and the IFN*γ*-inducible CXCR3 ligands. More recent studies have also suggested that CXCR3 plays a role in the migration of T cells in the microenvironment of the peripheral tissue and lymphoid compartment, facilitating the interaction of T cells with antigen presenting cells leading to the generation of effector and memory cells [26]. It has been reported [13] that Tregs accumulated in areas of inflammation in the liver of patients who had chronic hepatitis. These Treg cells expressed high levels of the chemokine receptor CXCR3. In a murine model of autoimmune hepatic inflammation [27], the CD4^+^CD25^+^Treg cells accumulated in the inflamed liver and this phenomenon was associated with the upregulation of CXCR3. These results provided strong evidence that CXCR3 is an important chemokine receptor that guides Treg cells into inflamed tissues and mediates local immune responses. However, whether CXCR3 is also important to the migratory function of Tregs in renal IRI remains unclear.

In our study, no CXCR3^+^ Tregs were detected in in the kidneys of normal mice. The subset significantly expanded in the IRI group at 72 h but not at 24 h after reperfusion. This observation demonstrated that CXCR3^+^ Tregs were not a resident population in normal kidney but accumulated in kidney after IRI. In addition, the inverse correlation of CXCR3^+^ Tregs with BUN and Scr levels as well as the tubular necrosis score in IRI mice was observed. This indicated that the increase in the number of CXCR3^+^ Tregs was associated with the recovery from kidney injury.

In the present study, we did not directly block CXCR3 to inhibit the migratory function. CXCR3 is expressed not only on helper T cells, but also on effector T cells and dendritic cells. The inflammatory CXCR3-chemokine dependent amplification loop also exits in hypoxia-reperfusion injury [28, 29]. Blocking CXCR3 would also inhibit the migratory function of inflammatory cells into the kidney and influence the process of IRI.

On the other hand, it should be noted that only 40% of Tregs in the kidney expressed CXCR3 after reperfusion in the present study. There might be other chemokines that also had the effect of recruiting Tregs into inflammatory tissue. Hoerning et al. [30] found not only CXCR3 but also CCR5 was related to the trafficking function of Tregs in renal transplantation in mice. Although further investigation is needed, our results still support CXCR3 as an important chemokine for the migratory function of Tregs in renal IRI.

## 5. Conclusions

In summary, the expanded Tregs participated in the repair of the early phase of renal IRI. CXCR3 might be an important chemokine receptor involved in the migration of Tregs into kidney tissue to serve an immunosuppressive function. These data revealed new insights into the pathogenesis of ischemic acute renal failure and suggest potential novel therapeutic approaches.
